# A prospective study comparing the efficacy and safety of two sublingual birch allergen preparations

**DOI:** 10.1186/2045-7022-4-23

**Published:** 2014-07-23

**Authors:** Ludger Klimek, Annette Sperl, Esther van Twuijver, Ronald van Ree, Huub Kleinjans, Johan Diderik Boot, Oliver Pfaar

**Affiliations:** 1 Center for Rhinology and Allergology Wiesbaden, Department of Otorhinolaryngology, Head and Neck Surgery, University Hospital Mannheim, Wiesbaden, Germany; 2 HAL Allergy BV, Medical Department, Leiden, The Netherlands; 3 Department of Experimental Immunology, Academic Medical Center, University of Amsterdam, Amsterdam, The Netherlands

**Keywords:** Birch pollen allergy, Sublingual immunotherapy (SLIT), Randomized, Non-inferiority design, Nasal provocation test (NPT)

## Abstract

**Background:**

SUBLIVAC FIX Birch (SUB-B) is a liquid oral preparation of *Betula verrucosa* pollen extract for the treatment of allergic rhinitis/rhinoconjuctivitis induced by birch pollen. The major allergen content of SUB-B and Staloral Birch (Stal-B) have been shown to be comparable. In order to compare the clinical efficacy and safety of both products, the present study was designed to investigate efficacy of treatment with SUB-B compared to Stal-B by means of reduction in allergy symptoms assessed by a titrated nasal provocation test (TNPT) in subjects suffering from IgE mediated allergy complaints triggered by birch pollen.

**Methods:**

A prospective, randomized, open, blinded endpoint (PROBE), controlled, single-centre study in 74 birch allergic adults was performed. Treatment consisted of either SUB-B (10,000 AUN/ml) or Stal-B (initial phase 10 I.R./ml and maintenance phase 300 I.R./ml) for 16–20 weeks at maintenance dose. The primary efficacy outcome was defined by the difference in change of the TNPT-threshold dose between the two treatment groups at baseline and after completion of treatment. Secondary outcomes included determination of birch pollen specific IgE and IgG levels, safety lab and ECG. During the first 30 days of treatment, subjects were requested to fill out a diary concerning compliance with study medication, occurrence of AEs and the use of concomitant medication.

**Results:**

Analysis of the primary efficacy parameter showed that the percentage of subjects showing a beneficial treatment effect was similar in both treatment groups, 33.3% for SUB-B vs. 31.4% for Stal-B in the intention to treat population. Evaluation of the immunologic response, showed that treatment with SUB-B and Stal-B induced similar increases (approximately 2 times) in IgE, IgG and IgG_4_ specific for Bet v 1.

In total, 143 related adverse events (AEs) were reported. The majority of the AEs was of mild intensity. The same pattern of AEs was observed for both products. No clinically relevant changes in other safety parameters, such as safety laboratory parameters, vital signs, physical examination and ECGs were observed.

**Conclusion:**

Taken together, treatment with both products was effective by means of reduction in allergic symptoms during a TNPT. In addition, safety analysis revealed a good tolerability of both SLIT extracts.

## Introduction

Allergic rhinitis is a global health problem, affecting all ethnic groups and ages and is estimated to affect 10-25% of the population [1,2]. Allergic rhinitis/rhinoconjunctivitis may significantly impair social life, school performance, work productivity and sleep in both adults and paediatric populations [1]. Moreover, if left untreated, allergic rhinitis/rhinoconjunctivitis is considered to be one of the major risk factors for the development of asthma [3-5].

Allergen specific immunotherapy is considered as an effective treatment for respiratory allergies. Specific immunotherapy is the only (causal) treatment modality with the capability of changing the natural course of the disease and thereby preventing its exacerbation and possible progression from rhinitis/rhinoconjunctivitis to asthma [4-6].

Sublingual immunotherapy (SLIT) is considered a viable and safe alternative to subcutaneous immunotherapy and is widely used in European countries [6,7]. The efficacy of SLIT in allergic rhinitis/rhinoconjunctivitis induced by various allergens, has been confirmed in multiple studies and, recently, sustained clinical effects, i.e. maintenance of significant and clinically relevant efficacy during two to three treatment years [8], of this form of SIT has been demonstrated [9-11].

The amount of allergen administered is crucial for both efficacy and safety of specific immunotherapy [11,12]. The major allergen in birch pollen is *Betula verrucosa* 1 (Bet v 1) and a recent study investigated the content of Bet v 1 in different SLIT products with a validated ELISA immunoassay [13]. The results showed that the amount of Bet v 1 (daily maintenance dose) in SUB-B and Stal-B was 46.7 μg and 25.4 μg respectively, both representing a high dose of major allergen [13]. Subsequently, the current study was performed to investigate the clinical efficacy and safety of SUB-B. Stal-B was chosen as a comparator product since this product is registered in Germany and has demonstrated efficacy in a previous clinical trial [14]. The primary objective of the study was to show, on an exploratory basis, that treatment with SUB-B is non-inferior to Stal-B by means of reduction in allergy symptoms assessed by TNPT in subjects suffering from IgE mediated allergy complaints triggered by birch pollen. Additionally the effect of treatment on allergen specific immunoglobulins was assessed. Furthermore, data with regards to safety of the product were obtained.

## Materials and methods

### Subjects

Seventy-four subjects with allergic rhinoconjunctivitis due to birch pollen with or without mild intermittent asthma (controlled by ß_2_ agonist use only) were included. A positive SPT (diameter ≥3 mm) for birch pollen (HAL Allergy B.V., Leiden, The Netherlands), a positive specific serum anti birch IgE test (>1 U/ml) and a positive TNPT with a birch pollen extract (HAL Allergy B.V., Leiden, The Netherlands) containing a concentration of 10, 100 or 1,000 AU/ml before start of treatment were required and were determined at the study site (Center for Rhinology and Allergology Wiesbaden). The main exclusion criteria were clinically relevant symptoms due to perennial allergies, chronic asthma or emphysema, with an FEV_1_ < 70% of predicted value, allergen specific immunotherapy within the past 5 years, completed or ongoing anti-IgE therapy, pregnancy, chronic or malignant diseases, drug or alcohol abuse and psychiatric disorders.

Written informed consent was obtained from all subjects. The study protocol was approved by the Ethik-Kommission der Landesärztekammer Hessen, Frankfurt-am-Main, Germany (approval no. FF 24/2009). The study was conducted in compliance with the latest version of the Declaration of Helsinki (DoH/Oct2008).

### Study design

The study was designed as a prospective, randomized, open, blinded endpoint, controlled, single centre study in Germany. The first patient was screened on 13 July 2009 and the last patient completed the study on 02 March 2010. Patients initiated treatment from 07 August 2010 until 23 October 2010, treatment duration was 16 weeks on average. All patients finished the study before the start of the birch pollen season, so it can be seen as a “pre-seasonal” schedule. Outcome measures were determined outside the birch pollen season, which started at the end of March 2010 in Germany. Due to the unavailability of Stal-B placebo, a blinded treatment using a three-arm, double-blind, double-dummy placebo-controlled design was not feasible. However, the assessment of the primary parameter was blinded, i.e. the assessor of the TNPT was not informed on the treatment group of subjects, enabling an independent assessment of the test. The assessor confirmed blinding by signing a statement of independent assessment.

Subjects were randomized to receive either SUB-B or Stal-B at a 1:1 ratio. SLIT consisted of either SUBLIVAC FIX Birch (10,000 AUN/ml; HAL Allergy BV, Leiden, The Netherlands) or Staloral Birch (10 I.R./ml and 300 I.R./ml; Stallergenes S.A., Antony, France), taken once daily and was started at the baseline visit. Treatment started with an up-dosing phase lasting 5 days for SUB-B and 9 days for the Stal-B preparations (6 days with vial 10 I.R./ml and 3 days with vial 300 I.R./ml). Thereafter the maintenance phase started and treatment was continued at a constant dose, from day 6 at 5 drops for SUB-B, and from day 10 at 4 puffs for Stal-B (vial 300 I.R./ml) for 16–20 weeks. The amount of Bet v 1 in the maintenance dose of both products was recently quantified with a validated ELISA immunoassay using Bet v 1 monoclonal antibodies (INDOOR Biotechnologies Ltd., Cardiff, UK).

During the first month of treatment, subjects were requested to fill out a diary concerning compliance with study medication, occurrence of AEs and the use of concomitant medication. Subjects visited the study centre before (screening and baseline visit), 1 month and 16–20 weeks after start of treatment (end of study). Vials of SLIT were returned to check compliance after four weeks and at the end of the trial.

### Assessment of efficacy

The titrated nasal provocation test (TNPT) was chosen as primary efficacy parameter. The TNPT-threshold dose was assessed at start of the study and after completion of treatment for each subject for both treatment groups, based on the German position paper on nasal allergen-challenge [15]. The procedure is briefly described as follows: before the provocation test subjects were acclimatized for 30 minutes in the test room. Rhinoscopy was performed prior to the measurement. Both the sum of reaction scores and nasal flow were assessed. Reaction scores of secretion (score 0–2), irritation/sneezing (score 0–2) and systemic symptoms (score 0–2) were determined (maximum score: 6). As an objective parameter, nasal airflow was additionally measured using rhinomanometry (MasterScopeRhino, CardinalHealth, Hoechberg, Germany). Flow rates at 150 Pa were obtained for both nostrils (ml/s). Sequential (allergen) dilutions were sprayed into the nose, after approximately 10 minutes the flow in both nostrils and symptom scores were determined. If the flow was more than 40% lower than the flow of the same side obtained with the previous measurement or the sum of the reaction scores was more than 3, the TNPT was regarded positive.

The allergen-challenge was performed with a solution containing diluent, 10 AU/ml, 100 AU/ml and 1,000 AU/ml (10,000 AU/ml, HAL-Allergy, Leiden, The Netherlands) at start of the study until a positive result was obtained. At the end of the study the TNPT was repeated with diluent, 10, 100, 1,000 or 10,000 AU/ml until a positive result was obtained. Dilutions were made on the day of the test from the stock solution of 10,000 AU/ml and one batch was used for all provocation tests. For each subject a comparison of the first and second TNPT was made. The treatment effect was scored as beneficial if the threshold dose of the second TNPT was higher than that of the first TNPT (Table 1). If this was not the case, the treatment effect was scored as non-beneficial. If a second TNPT was not performed, the result of the first TNPT was used for replacement of the missing second test results (Last Observation Carried Forward (LOCF-)principle).

**Table 1:** Evaluation of treatment effect

**2**^**nd **^**TNPT**	**Positive at 10 AU/ml**	**Positive at 100 AU/ml**	**Positive at 1,000 AU/ml**	**Positive at 10,000 AU/ml**	**Negative at 10,000 AU/ml**
**1**^**st **^**TNPT**					
Positive at 10 AU/ml	**-**	**+**	**+**	**+**	**+**
Positive at 100 AU/ml	**-**	**-**	**+**	**+**	**+**
Positive at 1,000 AU/ml	**-**	**-**	**-**	**+**	**+**

### Immunological measurements

Blood samples for determination of specific IgE, IgG and IgG_4_ were taken at baseline (before start of treatment) and at the end of the study. Birch and Bet v 1 specific IgE and IgG_4_ and Bet v 1 specific IgG antibodies were determined by ImmunoCAP® (Thermo Scientific, Uppsala, Sweden). Sera were analysed according to the specifications of the manufacturer at the Department of Experimental Immunology, AMC, University of Amsterdam, The Netherlands.

### Safety measurements

For safety measurements, adverse events (AE) were monitored throughout the study. A 30 minute observation period after the first administration of study medication was incorporated to observe immediate AE’s [6]. During the first month of treatment, subjects kept a daily record of all reactions occurring after taking the study medication. Before start of immunotherapy and at the end of study blood samples for analysis of safety laboratory values were collected and an ECG was performed. At all visits, a physical examination of the nose and mouth was performed and vital signs were checked.

### Statistical analysis

All analysis were performed both on the intention-to-treat (ITT) and per protocol (PP) population.

For the primary analysis the proportions of subjects, showing a beneficial treatment effect , were compared between the two treatment groups. A non-inferiority approach was chosen, using a non-inferiority margin of 30%. For investigation of a difference in treatment success the following hypotheses were tested:

H_0_: πStal - πSubl ≥ 0.3 vs. H_1_: πStal - πSubl < 0.3, whereby π_Subl_ and π_Stal_ denote the proportions of subjects, showing a beneficial treatment effect after SUB-B and Stal-B treatment, respectively. The one-sided 97.5% confidence interval for the difference in proportions between the two treatment groups was determined. H_0_ would be rejected if the confidence interval lies completely below 0.3. The global α-level for this study was 0.025 one sided.

For the results of specific immunoglobulin levels, comparison of the treatment groups was performed using the Wilcoxon rank sum test (two-sided). Analogous to this, pre-post differences for specific immunoglobulin levels before and after treatment were calculated.

For safety measurement the rate of subjects with at least one AE was compared between groups using Fisher’s exact test. Regarding safety laboratory values and vital signs a baseline comparison between treatment groups was performed using Wilcoxon rank sum test. Furthermore, shift analyses to show changes before and after treatment for safety laboratory values, ECG, vital signs and physical examinations were performed. For the secondary outcome measures an α -level <0.05 was considered significant.

With a sample size of 28 subjects per group, using a one-sided 0,025 significance level and non-inferiority margin of 30%, a power 80% was reached to reject the null hypothesis that the SUB-B and Stal-B are not equivalent.

## Results

### Study population

**Figure 1:**
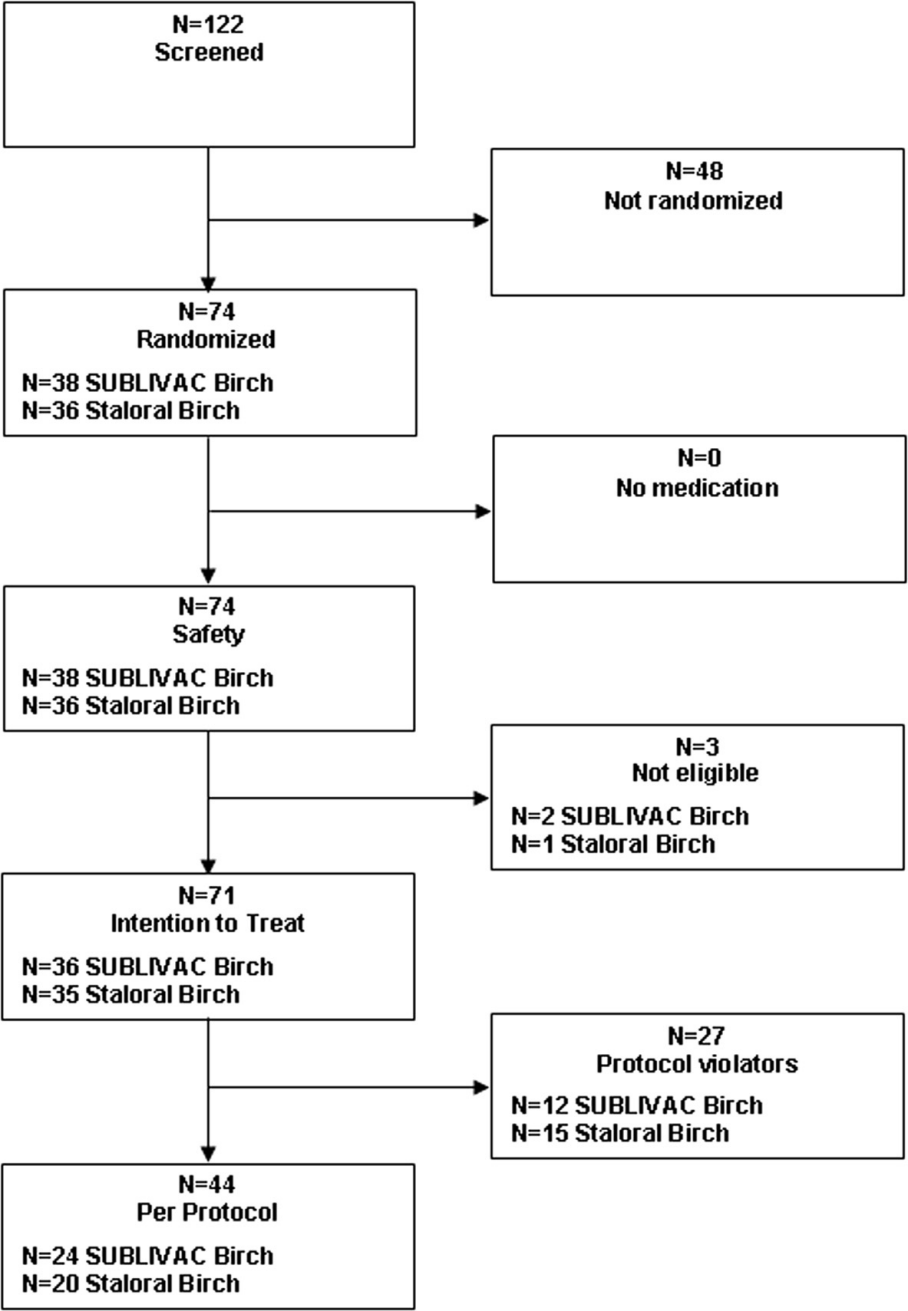
Disposition of patients.

A total of 122 subjects were screened and 74 subjects (safety population) were randomized and assigned to treatment with either SUB-B (n = 38) or Stal-B (n = 36).Three out of 74 subjects were assigned as not eligible and excluded from the analysis, since no post-baseline data were available (ITT population). In the ITT population the SUB-B treatment group consisted of 36 and the Stal-B treatment group of 35 subjects. Eight subjects terminated the study prematurely: 2 subjects were lost to follow-up, 1 discontinued the study due to adverse events with at an least ‘possible’ relationship to the study medication and 5 subjects withdrew informed consent (SUB-B n = 6; Stal-B n = 2; Figure 1).

Demographic data and baseline characteristics for both the ITT and PP population are provided in Table 2.

**Table 2:** Demographic and baseline characteristics of the study population

	**ITT population**		**Safety population**	
	**SUB-B**	**Stal-B**	**SUB-B**	**Stal-B**
Gender (% male/% female)	44.4/55.6	51.4/48.6	54.2/45.8	55.0/45.0
Age (years)	44.5 ± 11.6	45.1 ± 11.6	44.9 ± 11.3	45.6 ± 11.1
Body mass index (kg/m^2^)	25.5 ± 5.7	24.5 ± 3.8	26.7 ± 6.2	25.4 ± 4.4
Serum specific IgE to birch (U/ml)	32.5 ± 28.2	29.0 ± 24.8	Not determined	Not determined
Polysensitized patients (%)ª	75.0%	80.0%	76.3%	77.8%

Mean ± SD. ªSensitization to allergens other than tree pollen.

### Efficacy

**Figure 2:**
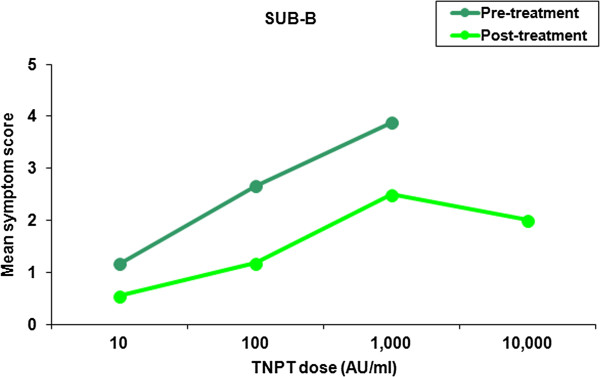
**Mean symptom scores following TNPT before and after treatment with SUB-B B (ITT population, n = 36).** The 10,000 AU/ml challenge was only given during the 2nd TNPT if the challenge at 1,000 AU/ml was negative.

**Figure 3:**
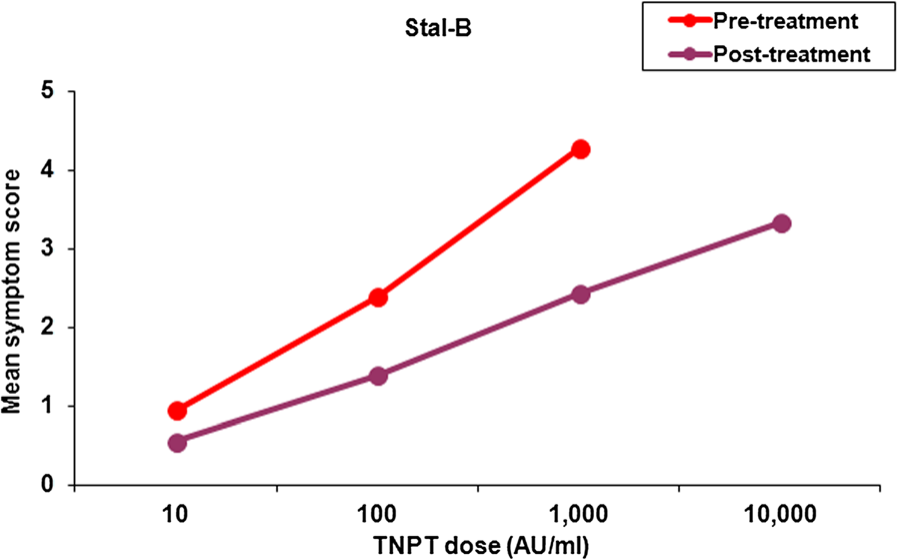
**Mean symptom scores following TNPT before and after treatment with Stal-B (ITT population, n = 35).** The 10,000 AU/ml challenge was only given during the 2nd TNPT if the challenge at 1,000 AU/ml was negative.

The change in the threshold dose of the titrated nasal provocation test before and after treatment with either SUB-B or Stal-B was determined per subject for both the ITT and the PP population. If the second TNPT was scored positive at a higher dose or even stayed negative at the highest dose tested, this indicated that subjects were less sensitive to birch pollen (=beneficial). The percentage of subjects showing a beneficial treatment effect was 33.3% (12/36 subjects) vs. 31.4% (11/35 subjects) in the ITT population and 45.8% (11/24 subjects) vs. 35.0% (7/20 subjects) in the PP population following SUB-B and Stal-B treatment, respectively. The results for both the ITT and PP population are displayed in Table 3. The one-sided 97.5% confidence interval for the difference in proportions between the two treatment groups (−1─0.181) lies completely below 0.3 indicating non-inferiority.Analogously, analysis of the symptom scores after nasal allergen showed a decrease of the mean symptom scores following both SLIT treatments in the ITT population (Figures 2 and 3). Not every patient received the same doses pre- and post-treatment (depended on the response). In addition, the 10,000 AU/ml dose was only administered at the end of the study. Therefore, no statistical analysis was performed on the mean decrease in overall symptom scores. However, if we compare the mean decrease in symptom scores over concentration 10–1,000 AU/ml a 45% vs. 43% decrease in symptom score was observed in the SUB-B and Stal-B treatment group compared to baseline, respectively.

**Table 3:** Treatment effect assessed by change in TNPT threshold dose following SLIT treatment

**Treatment**	**Intention-to-treat**			**Per-protocol**	
	**Beneficial (n)**	**Non beneficial (n)**	**Missing (n)**^**a**^	**Beneficial (n)**	**Non beneficial (n)**
**SUB-B**	33.3% (12)	47.2% (17)	19.4% (7)	45.8% (11)	54.2% (13)
**Stal-B**	31.4% (11)	54.3% (19)	14.3% (5)	35.0% (7)	65.0% (13)

^**a**^Incorrect assessment of baseline TNPT leading to violation of inclusion criteria.

In both treatment groups a similar decrease in flow reduction after each allergen challenge dose was observed following treatment.

### Specific immunological response

IgE, IgG and IgG_4_ specific for birch (Bet v) and Bet v 1 were determined per subject at the baseline visit (pre-treatment) and at the end of study visit (post-treatment) for both treatment groups.

**Figure 4:**
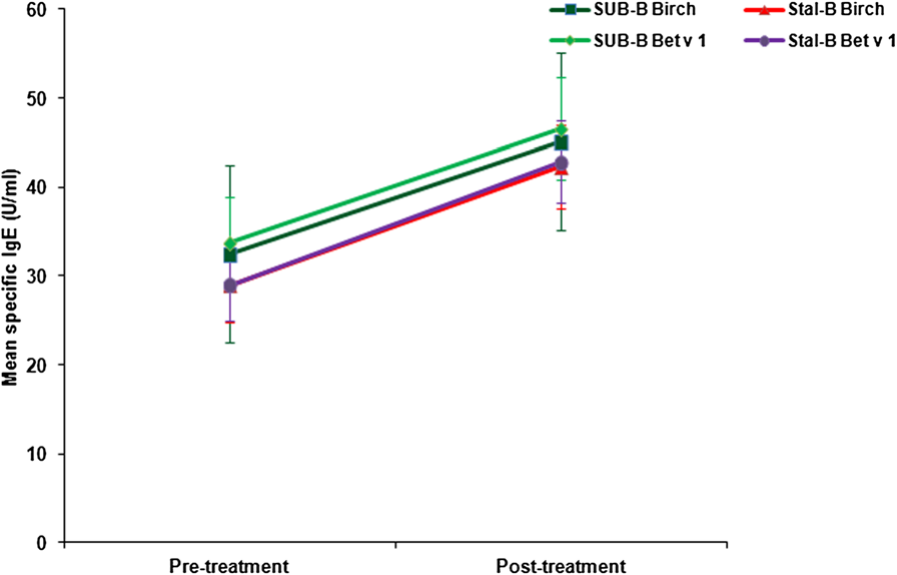
**Bet v and Bet v 1 specific IgE levels (including standard error) before and after SLIT treatment.** No significant differences in the increase in Bet v (p = 0.62) and Bet v 1 (p = 0.63) specific IgE levels was observed following SUB-B and Stal-B treatment.

**Figure 5:**
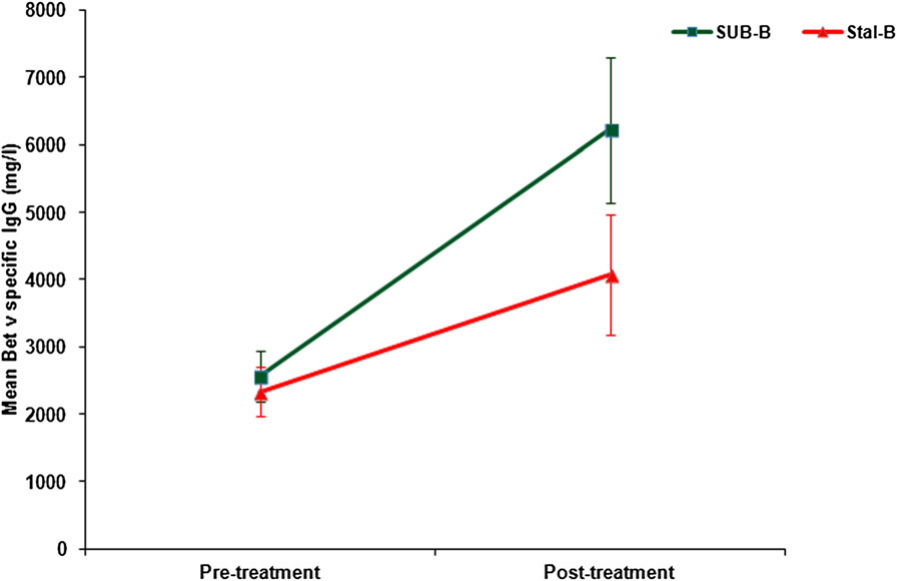
**Bet v specific IgG levels (including standard error) before and after SLIT treatment.** Bet v specific IgG levels increased in both groups, the increase in the SUB-B groups was significantly higher than in the Stal-B group (p = 0.03).

**Figure 6:**
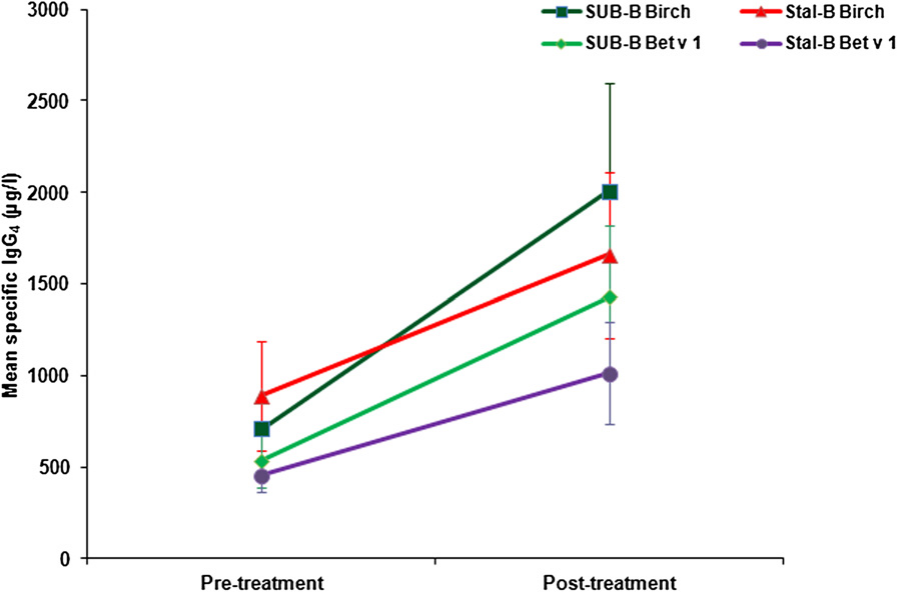
**Bet v and Bet v1 specific IgG**_**4 **_**levels (including standard error) before and after SLIT treatment.** No significant differences in the increase in Bet v (p = 0.17) and Bet v 1 (p = 0.11) specific IgG4 levels was observed following SUB-B and Stal-B treatment.

A comparable increase in birch (Bet v) and Bet v 1 specific IgE was observed in both treatment groups (Figure 4). Particularly specific IgG (Figure 5) and IgG_4_ (Figure 6) levels increased significantly (between 1.8 and 2.8 times) after treatment in both groups. Apart from Bet v specific IgG levels, no significant differences were detected in the increase of immunoglobulin levels between SUB-B and Stal-B treatment. Similar results were obtained in the PP population (data not shown).

### Safety

Treatment was well tolerated in both treatment groups, no unexpected AEs were observed. In total, 75.7% of all subjects (56/74) experienced a treatment-emergent AE, 28/38 (73.7%) treated with SUB-B and 28/36 (77.8%) treated with Stal-B. There was no significant difference in number of subjects reporting AEs between both treatment groups (p = 0.80).

Of the 164 AEs, 143 were related to the study medication (SUB-B n = 79, Stal-B n = 64). The majority of the related AEs was of grade I (mild) intensity in both treatment groups. The most common reported AEs were pruritus/swelling of mouth, tongue or lip (SUB-B: 65.8% of subjects; Stal-B: 61.1% of subjects). The pattern of AEs observed for both SLIT treatments was similar (Table 4). Symptom/sign designation was performed according to WAO grading of local and systemic adverse events [16-18].

**Table 4:** Overview most frequent related AEs (safety population)

**Symptom/sign**^**a**^		**SUB-B**			**Stal-B**		
	**Grade**	**No. of symptoms**	**No. of subjects**	**% of subjects**	**No. of symptoms**	**No. of subjects**	**% of subjects**
**Local reactions**							
Pruritus/swelling of mouth, tongue or lip	I	44	24	63.2	31	22	61.1
	II	1	1	2.6	0	0	0
Throat irritation	I	4	3	7.9	5	2	5.6
Ear pruritus	I	4	4	10.5	3	1	2.8
Nausea	I	3	2	5.3	0	0	0
Abdominal pain	I	3	3	7.9	1	1	2.8
**Systemic reactions**							
**Upper respiratory**							
Rhinitis (e.g., sneezing, rhinorrhea, nasal pruritus, and/or nasal congestion)	I	10	5	13.2	8	6	16.7
Cough	I	2	2	5.3	1	1	2.8
**Conjunctival**							
Conjunctival erythema, pruritus , or tearing	I	2	2	5.3	3	2	5.6
**Other**							
Headache	I	1	1	2.6	3	2	5.6

^**a**^Symptom/sign designation according to WAO grading of local and systemic adverse events [16-18].

During the study, one SAE was reported, which was assessed as not related to the study medication. Furthermore, no clinically relevant changes in safety laboratory parameters, vital signs, physical examination of the mouth and nose, and ECGs were observed and no statistical significant differences in these parameters between treatment groups were detected.

## Discussion

It has been acknowledged that the clinically effective doses of allergens for SLIT must be higher than for SCIT [1]. The amount of allergen administered is crucial for both efficacy and safety of specific immunotherapy and dose dependency has been reported [12]. The present study was designed to investigate the clinical efficacy and safety of SUB-B, containing a high dose of the birch pollen major allergen Bet v 1. Analysis of the results showed that the percentage of subjects showing a beneficial treatment effect was 45.8% vs. 35.0% in the PP population and 33.3% vs. 31.4% in the ITT population following SUB-B and Stal-B treatment, respectively. Since no significant differences between both treatment groups were observed, non-inferiority of SUB-B compared to Stal-B was concluded. Evaluation of the immunologic response, showed that treatment with both products induced a similar increase in IgG and IgG_4_ specific for Bet v and Bet v 1 (approximately 2 times). Moreover, non-inferiority was also shown for the treatment effect on the immunological response. In addition, safety analysis shows that both products are safe and cause mainly mild (grade I) and transient AEs.

The high and unpredictable variation in pollen levels and exposure to pollen may hamper the assessment of the efficacy, therefore it was decided to perform an exploratory comparison with a registered product (Stal-B), based on a surrogate parameter (TNPT). We chose the TNPT, since the response to nasal challenges has been shown to correlate to symptom-medication scores during the season and provocation tests are accepted endpoints in dose range finding studies [8,19]. In addition, nasal provocation tests can be combined with objective evaluation parameters such as rhinomanometry or peak nasal inspiratory flow (PNIF) [20]. In this single-centre study rhinomanometry was chosen, in multi-centre studies PNIF is a more suitable parameter. The reduction in TNPT response found in this study is therefore likely to be clinically relevant and translate into a decrease in seasonal symptoms for both allergen-preparations though this trial was not designed to investigate seasonal clinical efficacy by analyzing combined symptom-medication scores [8].

Although no differences between both treatment groups were observed, the percentage of subjects showing a beneficial treatment effect in the TNPT, i.e. an increase in the provocative threshold dose, was expected to be slightly higher in both treatment groups. This might be explained by the short treatment duration (on average 16 weeks on maintenance dose).

From the specific IgG assessments, a two-fold increase in specific IgG/IgG_4_ concentrations was observed in both treatment groups, supporting the immunogenic effect of both products. These results are comparable with the increase in IgG levels reported after sublingual immunotherapy with Staloral Birch [21]. Similarly, in a study with grass pollen SLIT the increase was also approximately two-fold after four months. However, the effect on the immunologic parameters was shown to be progressive with almost a fourfold increase after three treatment years [22]. Although the link between immunologic changes and clinical efficacy is not clarified as yet, this might suggest that the treatment duration in the present study was too short and longer treatment will result in maximal clinical and immunological effects.

Regarding safety, both the number of subjects experiencing AEs, and the frequency and nature of AEs were similar for both treatment with SUB-B and Stal-B. The majority of adverse reactions were of grade I (mild) intensity and all the patients fully recovered. Furthermore, no clinically relevant changes in safety laboratory parameters, vital signs, physical examination of the mouth and nose, and ECGs were observed. These results are comparable with results in other studies where SLIT has consistently shown to be safe and most adverse reactions are mild and occur during the induction phase of treatment both in paediatric patients and adults [6,23]. The most common adverse reactions are local in the oral mucosa and of the gastrointestinal system. Furthermore, during the study no clinically relevant changes in safety laboratory parameters, vital signs, physical examination of the mouth and nose, and ECGs were observed.

In conclusion, treatment with SUB-B was demonstrated to be as effective as treatment with Stal-B by means of reduction in allergic symptoms during nasal provocation in subjects suffering from IgE mediated allergic complaints triggered by birch pollen. In addition, SLIT with both preparations revealed a good safety-profile.

## Competing interests

LK and OP have received research grants from ALK-Abello, Denmark; Allergopharma, Germany; Stallergenes, France; HAL, The Netherlands; Artu Biologicals, The Netherlands; Allergy-Therapeutics/Bencard, UK/Germany; Hartington, Spain; Lofarma, Italy; Novartis/Leti, Germany/Spain; Glaxo-Smith-Kline, UK/Germany; Essex-Pharma, Germany; Cytos, Switzerland; Curalogic, Denmark; Roxall, Germany, Thermo-Fisher (Germany) and MEDA-Pharma GmbH (Germany). They have also served as advisors and on the speakers’ bureaus for some of the aforementioned pharmaceutical companies. OP has received travel grants from HAL Allergy, Netherlands/Germany, and Allergopharma, Germany and is a consultant for Bencard (Germany), HAL-Allergy (The Netherlands), Novartis (Germany), MEDA (Germany) and Stallergenes (France). OP is the current chairman of the EAACI Immunotherapy Interest Group and secretary of section ENT of DGAKI. EvT and DB are employees of HAL Allergy. HK was an employee of HAL Allergy when this study was performed. RvR has performed contract research for HAL Allergy and Stallergenes, and is consultant for HAL Allergy.

## Authors’ contributions

LK and OP were involved in the design of the study, acquisition and interpretation of data and in the drafting and revision of the manuscript. AS was involved in the acquisition and interpretation of data. EvT was involved in the design of the study, interpretation of data and in the drafting and revision of the manuscript. RvR was involved in the design of the study and revision of the manuscript. HK was involved in the design of the study and DB was involved in the interpretation of data and in the drafting and revision of the manuscript. All authors read and approved the final manuscript.
